# Oligomeric interfaces as a tool in drug discovery: Specific interference with activity of malate dehydrogenase of *Plasmodium falciparum in vitro*

**DOI:** 10.1371/journal.pone.0195011

**Published:** 2018-04-25

**Authors:** Sergey Lunev, Sabine Butzloff, Atilio R. Romero, Marleen Linzke, Fernando A. Batista, Kamila A. Meissner, Ingrid B. Müller, Alaa Adawy, Carsten Wrenger, Matthew R. Groves

**Affiliations:** 1 Structural Biology Unit, XB20 Drug Design, Department of Pharmacy, University of Groningen, Groningen, The Netherlands; 2 LG Müller, Bernhard Nocht Institute for Tropical Medicine, Hamburg, Germany; 3 Unit for Drug Discovery, Department of Parasitology, Institute of Biomedical Sciences, University of São Paulo, Saõ Paulo, Brazil; Universidade Nova de Lisboa Instituto de Tecnologia Quimica e Biologica, PORTUGAL

## Abstract

Malaria remains a major threat to human health, as strains resistant to current therapeutics are discovered. Efforts in finding new drug targets are hampered by the lack of sufficiently specific tools to provide target validation prior to initiating expensive drug discovery projects. Thus, new approaches that can rapidly enable drug target validation are of significant interest.

In this manuscript we present the crystal structure of malate dehydrogenase from *Plasmodium falciparum* (*Pf*MDH) at 2.4 Å resolution and structure-based mutagenic experiments interfering with the inter-oligomeric interactions of the enzyme. We report decreased thermal stability, significantly decreased specific activity and kinetic parameters of *Pf*MDH mutants upon mutagenic disruption of either oligomeric interface. In contrast, stabilization of one of the interfaces resulted in increased thermal stability, increased substrate/cofactor affinity and hyperactivity of the enzyme towards malate production at sub-millimolar substrate concentrations. Furthermore, the presented data show that our designed *Pf*MDH mutant could be used as specific inhibitor of the wild type *Pf*MDH activity, as mutated *Pf*MDH copies were shown to be able to self-incorporate into the native assembly upon introduction *in vitro*, yielding deactivated mutant:wild-type species. These data provide an insight into the role of oligomeric assembly in regulation of *Pf*MDH activity and reveal that recombinant mutants could be used as probe tool for specific modification of the wild type *Pf*MDH activity, thus offering the potential to validate its druggability *in vivo* without recourse to complex genetics or initial tool compounds. Such tool compounds often lack specificity between host or pathogen proteins (or are toxic in *in vivo* trials) and result in difficulties in assessing cause and effect—particularly in cases when the enzymes of interest possess close homologs within the human host. Furthermore, our oligomeric interference approach could be used in the future in order to assess druggability of other challenging human pathogen drug targets.

## Introduction

Malaria is a devastating disease and *Plasmodium falciparum* is responsible for the most lethal form of malaria [1]. During proliferation of the parasite within the host’s red blood cell the parasite depends on external nutrients that have to be imported and subsequently metabolized [2, 3]. This interconversion of nutrients is believed to be essential to provide the metabolic intermediates for the parasite’s growth. The validation of the druggability of these metabolic steps is highly challenging as the applicability of the probe techniques is limited in *P*. *falciparum* and depends on reverse genetics [4]. However, a possible alternative is offered through the examination of interaction surfaces between subunits of oligomeric proteins.

In recent publications Panchenko and colleagues summarised the biological importance, physico-chemical properties and evolutionary aspects of protein oligomerisation [5–7]. The authors showed that approximately 60% of the non-redundant protein structures available in the Protein Data Bank (PDB) are of dimeric or higher oligomeric order [6]. Protein oligomerisation is a feature shared by all organisms; oligomerisation is often essential to form functional protein complexes and can provide regulatory, stability, protective, selective, functional diversity or evolutionary benefits to the host organism [7]. Oligomeric interfaces also show extremely high specificity and binding affinity. In the majority of cases, oligomeric proteins can be purified from both recombinant and native sources in a single oligomeric state that show no non-cognate incorporation within the isolated assembly. Specificity of the oligomeric interfaces minimizes the interactions between unwanted partners, such as closely related and similar proteins of different pathways. Disruption of the native oligomeric state of the protein also often leads to the misregulation or the complete loss of function. Nishi and colleagues have shown a number of examples where mutations induce changes in the native oligomeric state, leading to devastating human diseases such as Alzheimer [7].

The evolutionary diversity of protein:protein interaction interfaces is also a key parameter in the study and treatment of infectious diseases. In many cases the proposed target protein from a pathogen shares function with a homologous protein of the human host. Tool compounds or methods that target the likely conserved active sites of the pathogenic enzymes will almost inevitably interact with the homologous proteins of the host system, as evolutionary constraints will restrict sequence diversity in these regions. Protein:protein interaction surfaces are less restricted by such constraints, as compensatory mutations can return the function of the surface (supporting oligomerisation) more diversely than mutations in an active site that must retain molecular function. This evolutionary diversity is also found in protein:protein interactions within oligomeric protein assemblies. Earlier studies have shown that protein interfaces tend to exhibit slightly higher level of evolutional conservation than the rest of the surface [8, 9] but significantly lower than that of active sites. Thus, we believe that oligomeric surfaces offer a potential in selective incorporation that may rival current cutting-edge genetic approaches to validate essential pathways for their druggability.

Enzymes within *plasmodial* carbon metabolism pathway have previously been suggested as promising targets for drug discovery [10, 11]. Particularly, our previous research was focused on *plasmodial* aspartate aminotransferase (*Pf*AspAT, **EC** **2.6.1.1**), an enzyme involved in aspartate metabolism, energy metabolism, pyrimidine biosynthesis as well as supplying the TCA cycle of the parasite with it’s intermediates [12–14]. Malate dehydrogenase (*Pf*MDH, **EC 1.1.1.37**) is located downstream of *Pf*AspAT in the cytosol of the parasite. It catalyzes the reversible reaction from malate to oxaloacetate using the reduction of NAD^+^ to NADH, thus aiding in the maintenance of the correct redox environment, crucial for the parasite’s survival particularly during the blood stages [15]. *Pf*MDH is also involved in the shuttle mechanism of the TCA cycle intermediates (malate/oxaloacetate), necessary for electron transfer from cytosolic NADH to the mitochondrial electron transport chain [16], a validated antimalarial drug target [17, 18]. These data make *Pf*AspAT as well as *Pf*MDH promising antimalarial drug targets.

Recently, the crystal structure of the *Pf*AspAT has been solved [13] and *Pf*MDH has been biochemically characterized and its spatial structure has been modeled [19–22]. Pradhan and colleagues reported a number of mutagenic experiments aimed at identification and characterization of key substrate and co-substrate binding pockets of *Pf*MDH [21]. Furthermore, based on their *in silico* model authors reported analysis of the quaternary structure of *Pf*MDH as well as attempts to disrupt its oligomeric assembly [20]. Despite these efforts, neither *Pf*AspAT nor *Pf*MDH has as yet been validated as a drug target. In both cases (as well as in case of other malarial enzymes that possess close homologs in human host) an inhibition tool with sufficient specificity *in vivo* is needed for successful drug target validation.

Here we report the crystal structure of *Pf*MDH at 2.4Å resolution. Based upon an examination of the crystal structure we have identified two major oligomeric interfaces within the tetramer and designed two point mutations (E18W and V190W) aiming at disruption of these interfaces. As anticipated, both mutations distorted the native oligomeric state, resulting in dimeric species with alternated kinetic parameters and significantly reduced specific activity. No evidence of significant misfolding could be derived, as both mutants retained basal activity and the ability to bind to oxaloacetate and cofactor NADH. Alternatively, introduction of additional hydrogen bonds via the E18Q mutation has likely stabilized the respective interface and resulted in increased specific activity of the enzyme (malate formation) at sub-millimolar substrate concentration.

Furthermore, our co-purification experiments showed that deactivated *Pf*MDH-V190W species were able to incorporate into the native *Pf*MDH assembly *in vitro*. The resulting chimeric protein contained both wild type and mutant copies, as confirmed by western blotting, and possessed no measurable activity.

These findings show that interference with oligomeric interfaces of *Pf*MDH could be used to modulate its function with high specificity. The structural information on *Pf*MDH has been used to generate recombinantly expressed mutant protein that could incorporate into the native assembly, inhibiting the specific activity *in vitro*. Extension of such a method *in vivo* could be used as a general tool approach in drug target validation, especially in challenging cases where the active site of the target protein is highly conserved or no tool compound with sufficient specificity is available.

## Results

**Fig 1:**
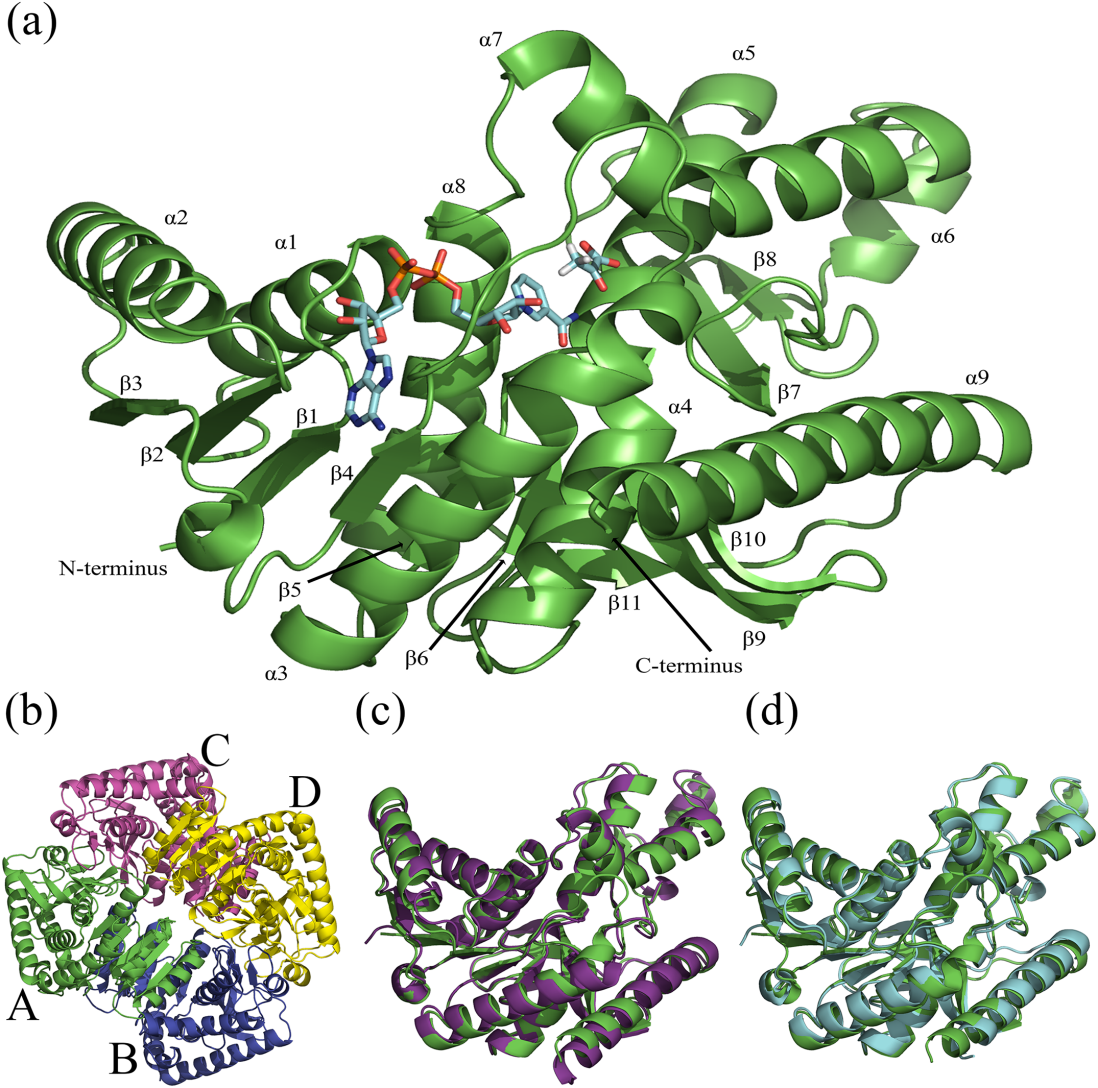
Fig 1 (a) shows the secondary structure of wild type *Pf*MDH as well as substrate and cofactor binding sites. Like other malate dehydrogenases, *Pf*MDH consists of 9 *alpha*-helixes and 11 *beta*-sheets. First 6 *beta*-sheets form parallel structure (Rossman fold) and belong to the cofactor-binding N-terminal domain. The NADH and pyruvate molecules were modeled using superposition with homologous MDH structure (**4PLC**, rmds 1 Å) [26]. (b) *Pf*MDH is a globular homo-tetramer, the subunits are labeled A, B, C and D. (c) Structural superposition of *Pf*MDH (green) with predicted ancestral apicomplexian malate dehydrogenase (53% sequence identity, 1 Å rmsd on C-*alphas*, **4PLC**) [26] shown in magenta. (d) Structural superposition of *Pf*MDH (green) with MDH from *Cryptosporidium parvum* (43% sequence identity, 1.3 Å rmsd on C-*alphas*, **2HJR**) [27] used for molecular replacement (cyan). Structures were superimposed using GESAMT [48] package from CCP4 suite [44] and visualized using PyMol [49].

Wild-type malate dehydrogenase (**EC 1.1.1.37**) from *Plasmodium falciparum* (*Pf*MDH-WT) was cloned, recombinantly expressed, purified and crystallised, as previously described [23]. *Pf*MDH-WT crystals belonged to P1 space group and diffracted to 2.4 Å. Molecular replacement yielded a clear solution of 4 tetramers in the asymmetric unit with R/R_free_ of 0,25/0,26, respectively (Table 1). The final model has been deposited in PDB under accession code **5NFR**. *Pf*MDH is a globular tetrameric protein [20, 23], where each monomer is comprised of 326 residues, which form 9 *alpha* helixes and 11 *beta*-sheets (Fig 1a and 1b). Similarly to other NAD-dependent dehydrogenases, the active sites are located in the cleft between two domains: an N-terminal cofactor-binding domain containing a parallel structure of first six *beta*-sheets (Rossmann-fold) and C-terminal substrate-binding domain (Fig 1a) [21, 24].

**Table 1:** Data collection and refinement statistics of *Pf*MDH.

	*Pf*MDH (5NFR)	
**Data collection**		
Beamline	X13 (EMBL)	BM14 (ESRF)
Space group	P1	P1
Cell dimensions:		
a, b, c (Å)	71.8, 156.6, 158.6	72.02, 152.69, 158.39
α, β, γ (o)	104.6, 101.1, 95.1	103.77, 101.46, 94.93
Resolution (Å)	19.76–2.95	47.6–2.4
Rmerge	17.6 (64.8)	5.3 (45.6)
Mean I/σI	5.88 (1.42)	10.46 (1.57)
Completeness (%)	92.0 (73.6)	89.5 (64.3)
Redundancy	2.59 (2.21)	2.12 (1.90)
**Refinement**		
Resolution (Å)		2.4
No. reflections		230493
*R*_*work*_ */ R*_*free*_		0.25 / 0.26
No. atoms:		
Protein		38114
Non-protein atoms		195
Average B-factors		
Protein (Å^2^)		23.49
Ions (Å^2^)		24.3
R.m.s. deviations:		
Bond lengths (Å)		0.019
Bond angles (°)		1.743
Ramachandran plot:		
Most favored, %		97.40
Allowed, %		2.27

*R*-factor is defined as (Σ_*hkl*_|*F*_*obs*_(*hkl*) − *F*_*calc*_(*hkl*)|)/Σ_*hkl*_ *F*_*obs*_ (*hkl*), where *F*_*obs*_ and *F*_*calc*_ are observed and calculated structure factors of the reflection of *hkl*, respectively.

*R*_*merge*_ is defined as Σ_*hkl*_ Σ_*i*_ | *I*_*i*_(*hkl*) − < *I*(*hkl*) > | / Σ_*hkl*_ Σ_*i*_ *I*_*i*_(*hkl*), where I_i_(*hkl*) is the i^th^ intensity measurement of reflection *hkl* and <I(*hkl*)> is the average intensity from multiple observations.

*R*_*free*_ was calculated on the basis of a small subset (5%) of randomly selected reflections omitted from the refinement. Values in parentheses correspond to the highest resolution shell. Data collection statistics are derived from previously reported article [23]

Overall, *Pf*MDH shows high structural homology with other apicomplexian dehydrogenases available in the PDB [25]. When superposed with a structure of a predicted ancestral apicomplexian malate dehydrogenase (**4PLH**) [26], *Pf*MDH structure shows an rmsd on C-*alpha* positions of approx. 1 Å (Fig 1c). Similarly, superposition with *Cryptosporidium parvum* MDH (**2HJR**) [27] shows an rmsd of 1.3 Å (Fig 1d). Subsequently, sequence conservation of *Pf*MDH amongst closely homologous species was analyzed using BLAST [28]. Overall, the residues of *Pf*MDH showed a sequence conservation of 32% amongst this set of closest relatives, with the residues comprising the active sites possessed a sequence conservation of close to 100% (Table 2). The analysis of the oligomeric interfaces as measured by PISA [29] online server, revealed an total solvent accessible surface area of 3641 Å^2^ (25% of the total accessible surface area) where 15.8% of the residues are conserved (Table 2).

**Table 2:** Sequence conservation of *Pf*MDH across homologs and surface analysis.

	*Pf*MDH (5NFR)			
No. of residues	313			
Conserved residues (% of total number of residues)	99 (31.6%)			
* absolutely	24 (7.7%)			
: strongly	42 (13.5%)			
. weakly	33 (10.5%)			
Active site residues (% of active site residues)	6			
* absolutely	5 (84%)			
: strongly	1 (16%)			
. weakly	0			
Interface	AB	AC	AD	Total
Interface residues	51	37	13	101
Conserved residues	11 (21.7%)	4 (10.8%)	1 (7.7%)	16 (15.8%)
* absolutely	6 (11.7%)	0	0	6 (5.9%)
: strongly	3 (5.9%)	3 (8.1%)	1 (7.7%)	7 (6.9%)
. weakly	2 (3.9%)	1 (2.7%)	0	3 (2.9%)
Total ASA per monomer (Å^2^)	14290			
Interface	AB	AC	AD	Total
Buried ASA (% of total ASA), Å^2^	1852.9 (13.0%)	1372.1 (9.6%)	421.3 (2.9%)	3641 (25.5%)

A table indicating the sequence conservation across the different oligomeric interfaces of *Pf*MDH. Sequence conservation of *Pf*MDH amongst closely homologous species was analyzed using BLAST (4). Overall, the residues of *Pf*MDH show a sequence conservation of 31.6% amongst this set of closest relatives, while the residues comprising the active sites possessed a sequence conservation of 84% (Fig 1b, 1e and 1f). Analysis of the oligomeric interfaces using PISA [29] revealed an overall oligomeric surface of 3641 Å^2^, with contributions to the different oligomeric interfaces as shown above.

**Fig 2:**
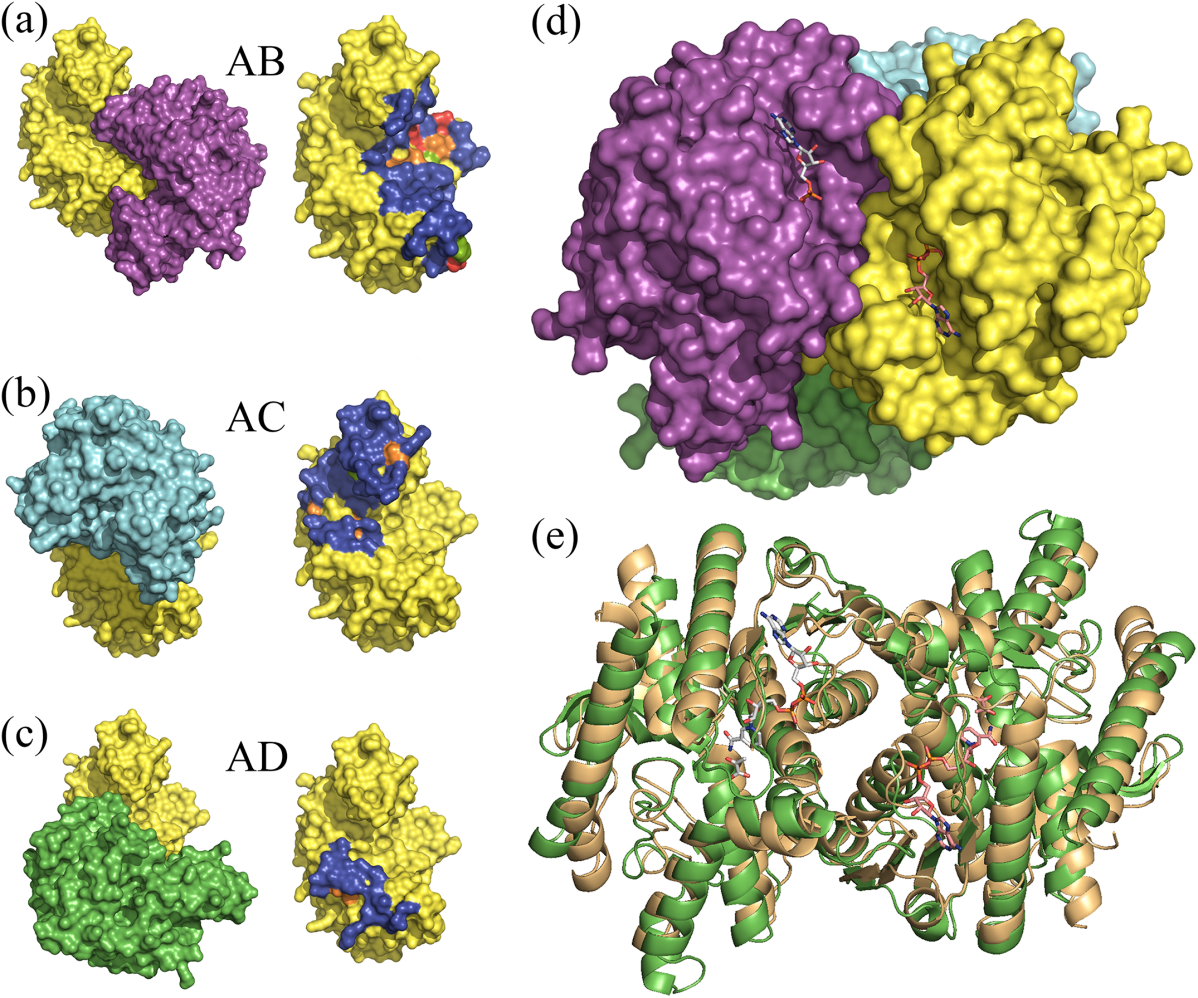
Figs 2 a-c show the interfaces formed between individual subunits of *Pf*MDH: AB (a), AC (b) and AD (c); residues involved in the oligomeric contact are shown in blue. Evolutionary conservation of the interface residues is shown in red (absolutely conserved), orange (strictly conserved) and green (slightly conserved). For more details on sequence conservation please refer to Table 2. (d) Positions of the active sites of adjacent subunits A (yellow) and B (Magenta) are shown. Active sites from A and B subunits are mirror reflections of each other, well separated and distal to AC interface. (e) Structural superposition of *Pf*MDH AB subassembly (green) and dimeric malate dehydrogenases from *E*. *coli* MDH (29% sequence identity, 2.5 Å rmsd, **2PWZ**, primary citation unavailable) shown in gold. In order to highlight the active site positions, the NADH and pyruvate molecules were modeled into the active sites using superposition with homologous MDH structure (**4PLC**, rmds 1 Å) [26]. Structure superposition was performed using GESAMT [48] package from CCP4 suite [44] and visualized using PyMol [49].

The oligomeric surfaces of *Pf*MDH can be split into two major groups: AB and AC (Fig 2a and 2b, Table 2). These surfaces comprise 1852.9 Å^2^ (AB) and 1372.1 Å^2^ (AC), respectively. The smaller interface (AD) likely does not represent a surface essential for oligomerization due to its relatively small size (421.3 Å^2^) and we have neglected it in the current analysis. Overall, 15.8% of the surface residues are conserved amongst the closest relatives. These observations agree with earlier reports, which show that in general enzyme oligomeric surfaces show significantly lower conservation than active site residues [8, 9]. Active sites from subunits A and B mirror each other and are located in close proximity to the AB interface (but well separated from each other and interface residues) (Fig 2d) and distal to the AC interface. The AB *Pf*MDH sub-assembly is highly similar to other dimeric malate dehydrogenases, such as *E*. *coli* MDH (29% sequence identity, 2.5 Å rmsd, **2PWZ**, primary citation unavailable) (Fig 2e). Structural comparison with Human type 2 MDH (28% identity; **2DFD**, primary citation not available) shows a lower degree of structural homology between the separate subunits of each tetramer (2.5 Å rmsd on C-*alphas*).

### Point mutations influence the oligomeric state of *Pf*MDH

**Fig 3:**
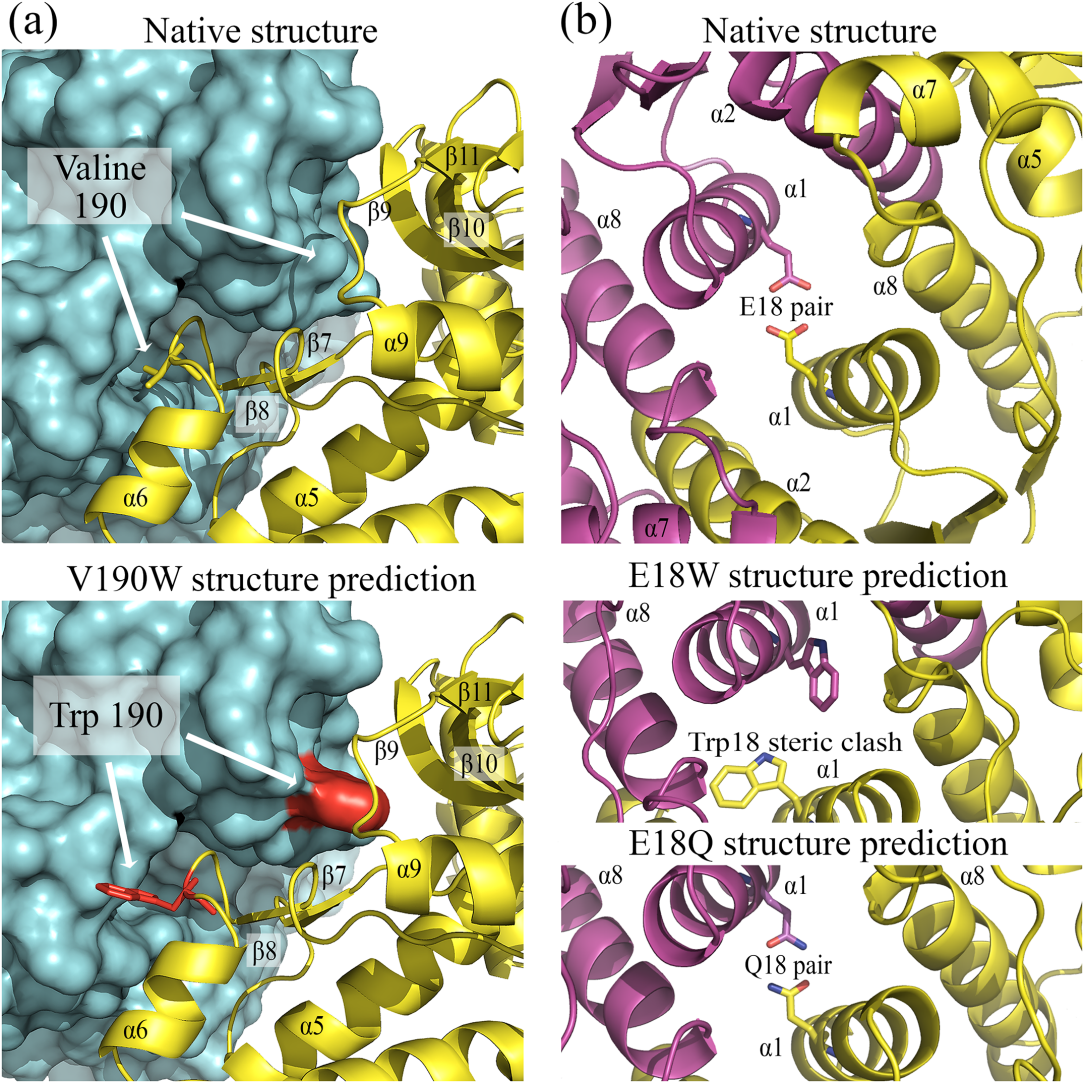
Fig 3. (a-top) Undisrupted oligomeric interface of *Pf*MDH (AC). Subunit A is schematically shown as cartoon (yellow), surface of the adjacent subunit C is shown in cyan. (a-bottom) Interface mutation V190W located between α6 and β8 causes disruption of the A-C interface as confirmed by SLS experiments. Point V190W mutations were modeled in *Pf*MDH structure using Coot [46] and visualized using PyMol [49]; mutated clashing residues are shown in red. (b-top) Native AB oligomeric interface of *Pf*MDH; Glutamate 18 pair in the core *α*1:*α*1 is shown in sticks. (b-bottom) Predicted steric clash caused by E18W mutation, causing an oligomeric disruption of AB interface and a model of the additional hydrogen bond pair (Gln18-Gln18) introduced between *α*1 helixes from adjacent subunits.

Based upon an examination of the crystal structure, point mutations of *Pf*MDH were designed to interfere with the AB and AC oligomeric surfaces. The loop region between *α*6 and *β*8 of each subunit (residues 187–192) was found reaching in the hydrophobic pocket region between *β*7 and *β*10 of adjacent subunit, facilitating the AC oligomeric contact (Fig 3a). The mutation V190W was designed to introduce a steric clash (Fig 3a) in that region of AC interface—potentially resulting in a dimeric form of *Pf*MDH similar to that observed in other organisms (e.g. *E*. *coli*; Fig 2e). The *Pf*MDH-V190W mutant was recombinantly expressed and purified. The impact of the mutations was confirmed by static light scattering measurements (Table 3), confirming that *Pf*MDH-V190W is dimeric in solution. These results have suggested that the mutation had the desired effect of disrupting the AC interface and that the AB interface in V190W mutant remained unperturbed, as this interface could still support dimerization.

**Table 3:** Summary of the biophysical parameters of *Pf*MDH mutants, as assessed by Static Light Scattering assay (SLS), Microscale Thermophoresis assay (MST) and Thermal Shift Assay (TSA).

*Pf*MDH	Wild type	V190W	E18W	E18Q
	**Static Light Scattering (SLS)**			
Oligomeric state	Tetramer	Dimer	Dimer	Tetramer
Molecular weight (kDa)	140.5 ± 4.2	70.5 ± 0.3	76.6 ± 0.4	139.4 ± 0.2
	**Microscale Thermophoresis (MST)**			
Dissociation constants:				
Oxaloacetate^&^	3.93 ± 0.58 mM	3.86 ± 0.80 mM	621 ± 220 μM	823 ± 33 μM
Malate^&^	Nd	Nd	Nd	Nd
NADH^£^	172 ± 73 μM	474 ± 105 μM	1.66 ± 0.61 mM	3.2 ± 1.2 μM
NAD^+^ ^£^	Nd	Nd	Nd	Nd
	**Thermal Shift Assay (TSA)**			
Samples	T_m_ (ΔT_m_ compared to Assay Buffer sample), K			
Assay Buffer	331	315	320	333.5
Oxaloacetate (20 mM*)	331 (0)	316 (1)	319 (-1)	333.5 (0)
Malate (20 mM*)	333 (2)	316 (1)	318 (-2)	333.5 (0)
NADH (10 mM*)	341 (10)	322 (7)	326 (6)	341 (7.5)
NAD^+^ (10 mM*)	334 (3)	318 (3)	319 (-1)	335,5 (0)

^&^ Assays performed in the absence of co-factors

^£^ Assays performed in the absence of substrates

* in Assay Buffer (100 mM Na-Phosphate pH 7.4 and 400 mM Na Cl)

Oligomeric state determination of the *Pf*MDH WT and its mutants was performed using static light scattering (SLS), as described in Materials and Methods section. Dissociation constants for NAD and Malate could not be determined for either WT or mutant versions of *Pf*MDH even at high substrate/cofactor concentrations reaching 20 mM. TSA experiments performed in the Assay buffer (100 mM Na-Phosphate pH 7.4, 400 mM NaCl) assessed the effects of 20 mM oxaloacetate, 20 mM malate, 10 mM NADH and 10 mM NAD^+^ on thermal stability of *Pf*MDH and its mutants.

The AB interface is mainly formed by residues that belong to *alpha* helixes 1, 2, 5 and 8 from both subunits. The core non-conserved region between the *α*1 helixes of each subunit is mainly hydrophobic with the exception of the acidic Glu 18 pair (Fig 3b). Similarly to the V190W case, a point mutation E18W was designed to introduce a steric clash between the subunits A and B (Fig 3b). *Pf*MDH-E18W mutant was recombinantly expressed, purified and subsequently characterized using SLS measurements (Table 3), confirming the presence of dimeric species.

The E18Q mutation was designed to strengthen the AB interface by replacing the Glu:Glu interaction with complementary hydrogen bonding pair between the mutated glutamines 18 from adjacent chains (Fig 3b). Similarly, *Pf*MDH-E18Q was recombinantly expressed and purified. The ability of the *Pf*MDH-E18Q mutant to form tetramers *in vitro*, as confirmed by SLS (Table 3), strongly suggests that there were no adverse alterations to the surfaces that support oligomerisation (either AB or AC).

### Thermal stability of *Pf*MDH interface mutants indicates the mutants adopt a well-folded conformation

**Fig 4:**
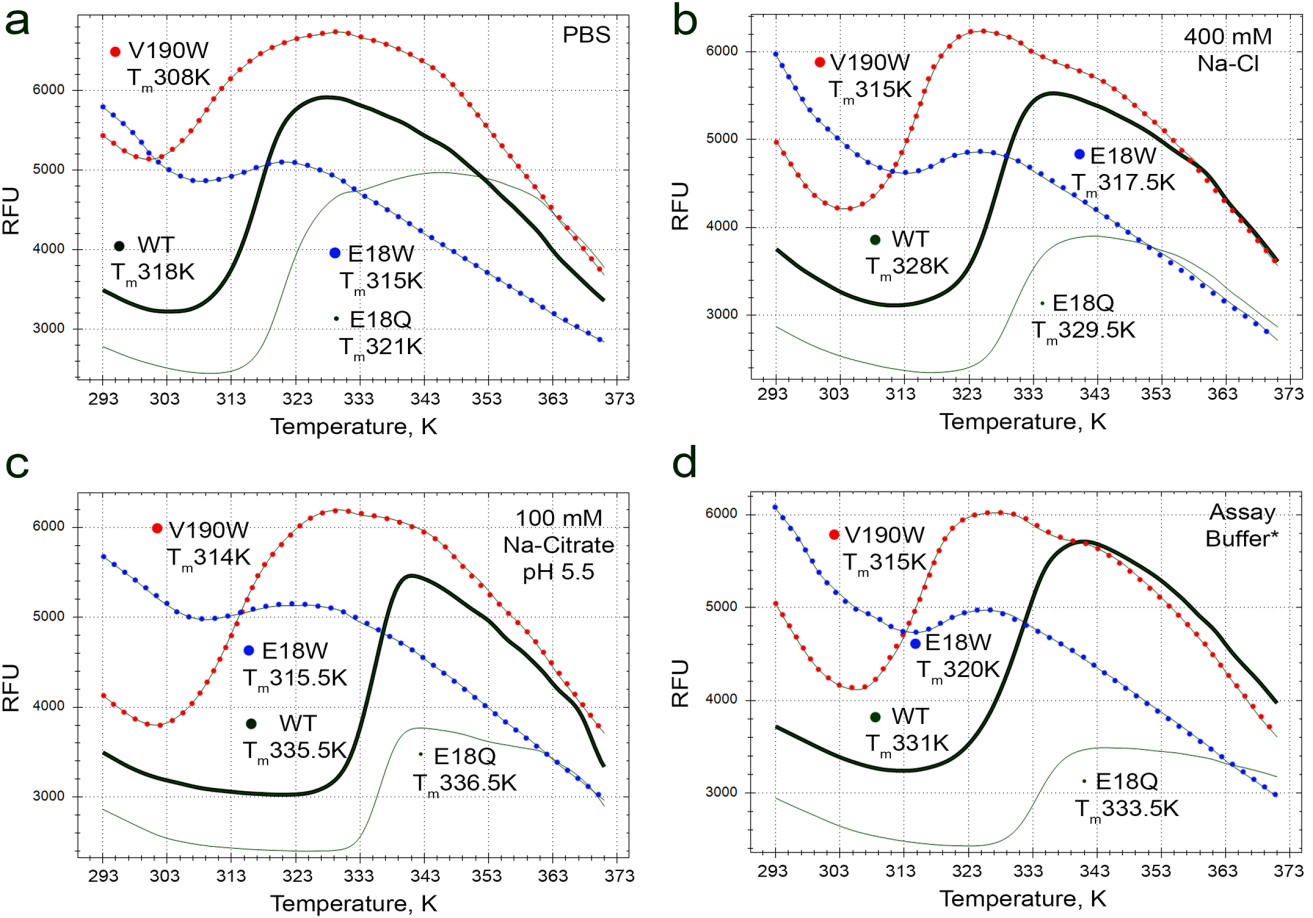
Fig 4 shows examples of TSA melting curves of *Pf*MDH WT (dark bold lines), *Pf*MDH-V190W (dotted red lines), *Pf*MDH-E18W (dotted blue lines) and *Pf*MDH-E18Q (faint lines) in various buffer conditions: (a) PBS, (b) 400 mM NaCl, (c) 100 mM Na-Citrate pH 5.5 and (d) Assay Buffer (100 mM Na-Phosphate pH 7.4, 400 mM NaCl). Melting temperatures of each sample are displayed next to the respective curves. Analysis of these curves shows that *Pf*MDH is rather unstable in PBS (a) and requires optimized buffer conditions for further experiments. This effect is more pronounced for its mutant forms, where native oligomeric assembly has been disrupted (dotted lines). *Pf*MDH-E18Q mutant shows higher thermal stability, thus supporting the hypothesis that introduction of the additional hydrogen bond pair at the AB interface has had the desired stabilization effect. (b) 400 mM NaCl has significantly stabilized the wild type *Pf*MDH (ΔT_m_ = 10 K), dimeric V190W mutant (ΔT_m_ = 7 K) and tetrameric E18Q mutant (ΔT_m_ = 8.5 K), while having minor effect of the E18W dimeric mutant (ΔT_m_ = 2.5 K). (c) 100 mM Na-Citrate pH 5.5 significantly stabilized the wild type enzyme (ΔT_m_ = 17.5 K) and the E18Q mutant (ΔT_m_ = 15.5 K), while having lesser effect on V190W mutant (ΔT_m_ = 6 K) and negligible effect on E18W (ΔT_m_ = 0.5 K). (d) Selection of the Assay Buffer allowed further experiments to be performed for all four *Pf*MDH constructs used in this study in the same stabilizing buffer conditions (WT ΔT_m_ = 13 K, V190W ΔT_m_ = 7 K, E18W ΔT_m_ = 5 K, E18Q ΔT_m_ = 12.5 K). TSA assays were performed in 96-well PCR plates (VWR) using SFX96 Real-Time PCR reactor (BioRad). Melting curve (in terms of increased fluorescence, RFU) of each sample was plotted against the temperature gradient (293–363 K) using BioRad SFX96 software and the temperatures of the inflection points (T_m_’s) were used as indicators of the thermal stability of each sample. ΔT_m_’s reflect stabilization effect of each condition compared to the control experiments performed in PBS. For more details refer to Materials and Methods section.

Purified *Pf*MDH WT as well as the mutant samples were assayed against a buffer-component library using thermal shift assay (TSA) [30–32]. Interestingly, all *Pf*MDH samples were rather unstable at physiological conditions (in PBS, Fig 4a), agreeing with previously reported sharp thermal transition and activity loss in the range of 313–318 K observed for *Pf*MDH, purified directly from *P*. *falciparum* parasites [22]. Taken together these data the buffer conditions for further assays have been optimized. Among tested buffer components, 100 mM Na-citrate pH 5.5 and 400 mM NaCl showed highest midpoints of the melt curves (interpreted as T_m_, Fig 4). As a result of TSA analysis the Assay Buffer used for further experiments consisted of 100 mM Na-phosphate and 400 mM NaCl at pH 7.4. Citrate was not used in the Assay Buffer due to the annotated activation behavior of citrate on MDH activity [33]. Possible effects of acidic as well as citrate-based buffer systems on *Pf*MDH have not been addressed in this study. Increased melting temperatures of *Pf*MDH-WT (ΔT_m_ = 13 K), V190W (ΔT_m_ = 7 K), E18W (ΔT_m_ = 5 K) and E18Q (ΔT_m_ = 12.5 K) samples in the Assay Buffer compared to PBS allowed the use of Assay Buffer for all four *Pf*MDH samples in further experiments.

Little or no stabilization effect was observed upon addition of 20 mM substrate (malate or oxaloacetate) to the Assay Buffer for all four *Pf*MDH samples (Table 3). However, the samples were significantly stabilized upon addition of 10 mM NADH cofactor (ΔT_m_ 6–10 K). Addition of other cofactor, NAD^+^ caused little or no effect on thermal stability of *Pf*MDH wild type and mutant samples (Table 3).

Interestingly, but not unexpectedly, disruption of the native oligomeric interface in case of both V190W and E18W resulted in significantly reduced thermal stability (T_m_’s approximately 10–15 K lower, Fig 4, Table 3) compared to the wild type enzyme. The *Pf*MDH-V190W mutant in which the AC interface was disrupted retained the preference for lower pH citrate buffer (ΔT_m_ = 6 K, Fig 4c) and high ionic strength (ΔT_m_ = 7 K, Fig 4b). Unlike V190W, E18W mutation disrupting the AB oligomeric interface resulted in significantly less stable enzyme species inert to either citrate (ΔT_m_ = 0.5 K) or high ionic strength (ΔT_m_ = 2.5 K)(Fig 4b and 4c). As anticipated, E18Q mutation introducing an additional hydrogen bond pair into the AB interface resulted in mild stabilization of the enzyme (ΔT_m_ = 1–3 K, Fig 4, Table 3).

Overall these data indicate that all *Pf*MDH mutants adopted a folded conformation, in which the hydrophobic core of the protein is shielded from the bulk solvent, as all three mutants demonstrated TSA-determined T_m_’s consistent with globular proteins, rather than denatured or unfolded proteins [32, 34]. The reduction in stability of *Pf*MDH-V190W and *Pf*MDH-E18W mutants compared to the wild type could be attributed to the stabilizing effects of tetramer assembly upon the individual monomer folds. Relatively high initial fluorescence signal observed for V190W and E18W samples (Fig 4) compared to the wild type enzyme could be explained by exposure of hydrophobic parts of AB and AC interfaces, usually shielded from the solvent, to the SYPRO orange dye.

### Oligomeric distortions influence the substrate/cofactor binding of *Pf*MDH

Microscale Thermophoresis (MST, Nanotemper GmgH) experiments performed with fluorescently labeled (see Materials&methods) *Pf*MDH-WT supported previously proposed mechanism, where the cofactor NADH/NAD^+^ must bind prior the substrate [24]. Indeed, the affinity measured for NADH binding (172 ± 73 μM, Table 3) was much higher that that of substrate oxaloacetate (3.9 ± 0.6 mM, Table 3). Distortion of the AC interface (distal from the active sites) via V190W mutation caused 3-fold decreased affinity (K_d_ 474 ± 105 μM) towards NADH and no apparent change in oxaloacetate binding (K_d_ 3.9 ± 0.8 mM). In contrast, AB interface distortion via E18W mutation resulted in 10-fold decreased NADH affinity (1.7 ± 0.6 mM) and unexpectedly 6-fold increased affinity towards oxaloacetate (0.62 ± 0.22 mM).

Stabilization of the AB interface via E18Q mutation resulted in significantly increased binding affinity towards both NADH and oxaloacetate (K_d_’s 3.2 ± 1.2 μM and 823 ± 33 μM, respectively; Table 3). Binding assays with substrates were performed separately. No measurable binding of NAD^+^ or malate could be observed for all *Pf*MDH constructs, supporting previous hypothesis that under standard conditions the reaction is more favorable towards malate formation [24].

### Oligomeric distortions influence the specific activity of *Pf*MDH

**Fig 5:**
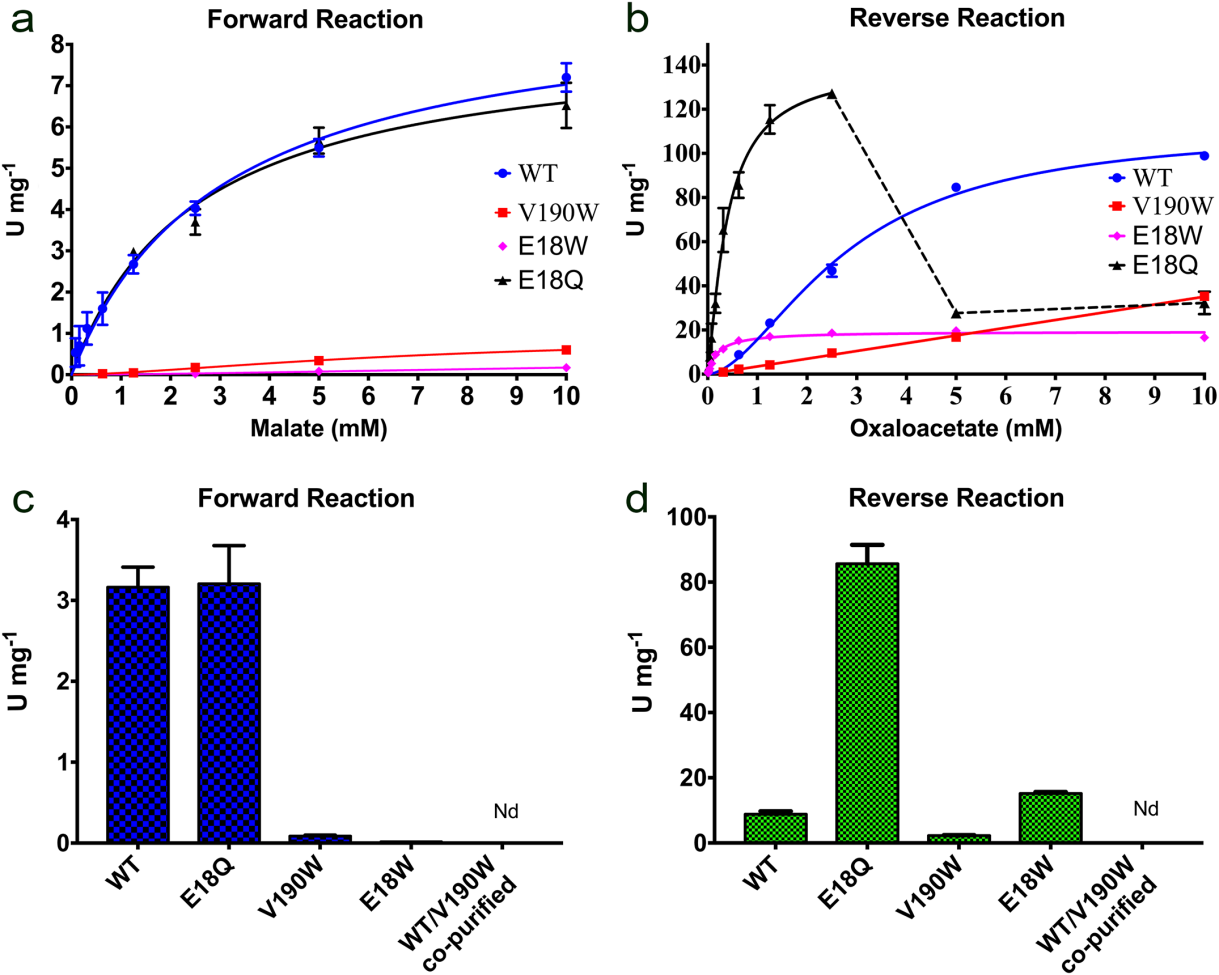
Fig 5 shows specific activity (both forward and reverse reactions) of the wild type *Pf*MDH, E18Q, V190W, E18W mutants, and co-purified WT-mutant chimeric complex. Specific activity is shown in Units per mg of enzyme (U mg^-1^). U = μmol of NADH oxidized or (NAD^+^) reduced per minute. (a & b) Interference with the native oligomeric state of *Pf*MDH affected substrate kinetics as well as significantly changed specific activity of the mutant species. (b) E18Q mutant showed increased specific activity for reverse reaction as well as significant substrate inhibition at substrate concentrations above 2.5 mM. (c) At 1.6 mM malate (reported intracellular substrate concentration [35]) dimeric *Pf*MDH mutants had significantly reduced specific activity towards malate oxidation, while E18Q mutant activity was not significantly changed compared to WT. No activity could be detected for co-purified *Pf*MDH-WT/V190W chimeric assembly. (d) At sub-millimolar substrate concentration (0.625 mM oxaloacetate) E18Q mutant showed significantly increased (10x) specific activity, while V190W mutant or co-purified WT/V190W chimera showed little or no measurable activity, respectively. Interestingly, E18W mutation disrupting AB interface resulted in slightly increased activity towards oxaloacetate reduction compared to the wild type enzyme.

Specific activity of the wild type *Pf*MDH and its mutants was measured in terms of amount of NADH oxidized or (NAD^+^) reduced (U = 1 μmol NADH min^-1^) per milligram of the enzyme. Wild type *Pf*MDH displayed Michaelis-Menthen kinetics for forward reaction (malate oxidation) with V_max_ of 9.2 ± 0.4 U mg^-1^ and K_m_ of 3.0 ± 0.3 mM of malate (Fig 5a). Reverse reaction (oxaloacetate reduction) was interpreted as positively cooperative with Hill coefficient of 1.75 ± 0.11, significantly higher V_max_ of 111 ± 4 U mg^-1^ and K_half_ of 2.8 ± 0.2 mM (Fig 5b). These data are consistent with previously reported specific activity of *Pf*MDH measured to be approx. 80 U mg^-1^ and no observed substrate inhibition at high oxaloacetate concentrations [22].

Distortion of the native oligomeric assembly via V190W and E18W mutations has significantly affected the enzyme activity and substrate kinetics. Indeed, forward reaction catalyzed by the dimeric *Pf*MDH-V190W mutant was interpreted as cooperative with Hill coefficient of 1.5 ± 0.1. Maximal measured specific activity was approx. 0.6 U mg^-1^ and calculated K_half_ was above 7 mM (Fig 5a). Reverse reaction followed nearly linear trend with maximal measured activity of 35.2 ± 1.1 U mg^-1^ at 10 mM oxaloacetate (Fig 5b).

Similarly, disruption of AB interface via E18W mutation resulted in significantly reduced maximal specific activity measured for forward reaction (approx. 0.17 U mg^-1^). The reaction could be interpreted as cooperative with Hill coefficient of 1.45 ± 0.3 (Fig 5a). Reverse reaction catalyzed by E18W mutant followed Michaelis Menten kinetics with V_max_ = 19.3 ± 0.4 U mg^-1^ and K_m_ = 0.20 ± 0.02 mM (Fig 5b).

Interestingly, E18Q mutation did not significantly change the forward enzyme activity. The reaction followed Michaelis-Menthen kinetics with slightly lower calculated K_m_ (8.3 ± 0.6 U mg^-1^) and reduced K_m_ (2.5 + 0.5 mM), compared to the wild type *Pf*MDH (Fig 5a). In contrast, reverse reaction of E18Q was interpreted as cooperative with Hill coefficient of 1.2 ± 0.1 (Fig 5b) at substrate concentrations below 2.5 mM oxaloacetate. Above that concentration E18Q mutant showed significant substrate inhibition (Fig 5b). V_max_ of the reverse reaction (below 2.5 mM oxaloacetate) increased to 140 ± 6 U mg^-1^ with significantly lower K_half_ of 0.38 ± 0.4 mM.

### Oligomeric interfaces can be used to incorporate deactivated mutants into a *Pf*MDH assembly after recombinant expression

**Fig 6:**
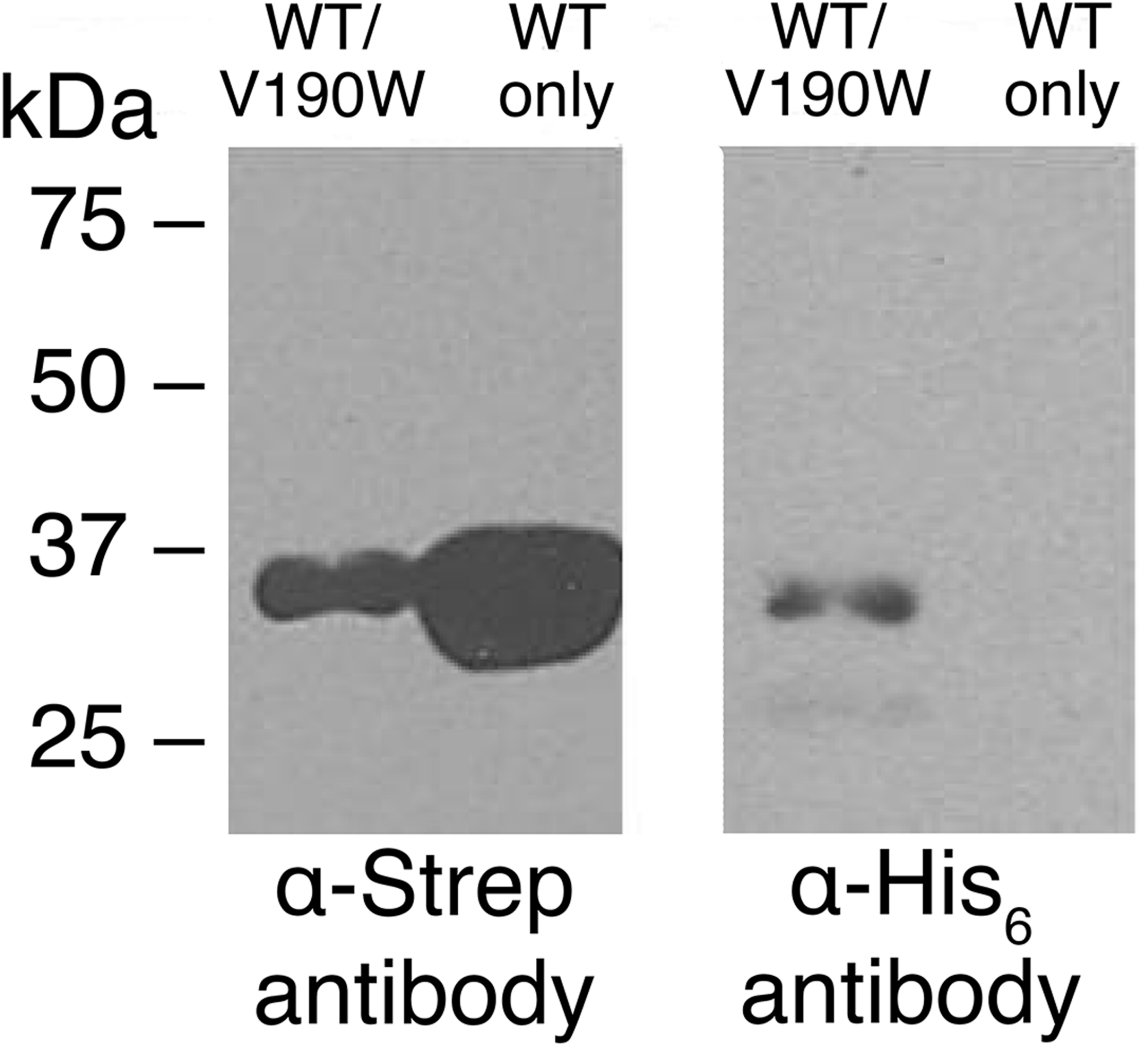
Fig 6. Western Blot analysis demonstrates the ability of His_6_-tagged *Pf*MDH-V190W mutants to incorporate into pre-formed native Strep-tagged oligomeric assembly post-expression. Both proteins were recombinantly expressed and the lysate were mixed and sequentially purified via Ni-NTA and Strep-tactin chromatography. (Left) Mixed lysate and wild-type *Pf*MDH were first purified via Strep-Tactin (IBA Lifesciences) chromatography and subsequently analyzed by Western Blot using α-strep antibodies (See Materials and methods), confirming the presence of Strep-tagged *Pf*MDH-WT in both samples. (Right) Strep-purified samples were further purified via Ni-NTA chromatography. Western Blot with α-His antibodies showed no signal for the wild type sample (as expected) and confirmed the presence of His_6_-tagged V190W mutant in co-purified sample.

Recombinantly expressed wild-type *Pf*MDH-WT (Strep-tagged) and *Pf*MDH-V190W (His_6_-tagged) were expressed separately in *E*. *coli*. The lysates were mixed and purified by sequential streptactin and Ni-NTA-affinity chromatography to isolate the wild-type:mutant complex. Subsequent Western Blot analysis demonstrated that *Pf*MDH-V190W was able to insert itself into a pre-formed wild-type *Pf*MDH assembly (Fig 6). Subsequent activity assays demonstrated that while recombinant wild-type *Pf*MDH displayed both reductive and oxidative activity, the isolated wild-type:V190W chimera possessed no activity in either direction (Fig 5c and 5d).

These data support our hypothesis that a properly formed oligomeric assembly is required for correct catalytic function of *Pf*MDH and that a chimeric assembly can be generated through introduction of dimeric *Pf*MDH-V190W species to the wild-type tetrameric *Pf*MDH *in vitro*. The chimeric *Pf*MDH:*Pf*MDH-V190W has likely a perturbed AC interface and is shown to be inactive in *in vitro* activity assays. In conclusion, deactivated oligomeric mutants can be used *in vitro* as specific inhibitors of *Pf*MDH activity.

## Discussion

The structural data agree with previous findings, in which *Pf*MDH was reported as homo-tetramer in solution [20]. While the majority of MDHs exists in a tetrameric form [24], a number of homologs from other species have been reported to exist in a dimeric form; and the oligomeric assembly of MDH has been suggested to be critical for the enzymatic function [20, 24]. Each monomeric subunit of *Pf*MDH is comprised of 11 *beta*-sheets and 9 *alpha*-helixes (Fig 1a). Overall the fold of *Pf*MDH is highly similar to those of previously determined MDHs (see Results section). While enzymes of the malarial carbon metabolism pathway have been suggested to be promising drug targets, the similarity in fold and associated similarity in position of the active site residues (Table 2) provides a further demonstration of the difficulties of validating MDH as a malarial drug target. An insufficiently specific inhibitor of the MDH active site would almost certainly have significant cross-reactivity with the human MDH homologs—leading to difficulties in deconvoluting the effect of the MDH inhibitor on the parasite in *in vivo* assays. Unfortunately, genetic approaches in the malarial parasite are non-trivial and would not necessarily provide a clearer route to the validation of *Pf*MDH as a drug target [4].

Analysis of the crystal structure of *Pf*MDH showed that AB assembly is highly similar to that of the active *E*. *coli* dimer (Fig 2e). Interestingly, the disruption of the AC interface via V190W mutation (Fig 3a and 3b) likely breaks the native tetrameric *Pf*MDH assembly into two inactive dimers (Fig 5a and 5d), as supported by SLS (Table 3). Previous attempts to interfere with the native tetrameric assembly of *Pf*MDH have been reported [20]. The authors proposed that disruption of a salt bridge near the AC oligomeric interface (*Pf*MDH-R183A/R214G) would cause the splitting of the tetramer into an AB dimer, analogous to the dimeric *E*. *coli* MDH. However, while this mutant retained activity, the investigation of the oligomeric state was performed using size exclusion chromatography rather than direct biophysical measurements. In our study, static light scattering experiments are used to demonstrate the oligomeric states of mutants, highlighting the importance of precise biophysical measurements when estimates of oligomeric state are made. Analysis of our crystal structure of *Pf*MDH also suggests that *Pf*MDH-R183A/R214G could potentially re-assemble into a tetramer in solution at least transiently, thereby retaining activity, as no inter-oligomeric clashes were introduced by these mutations. In contrast, our data demonstrate that the V190W and E18W interface mutations not only cause the breakdown of the native assembly into dimers (Table 3), but also make the transient re-formation of the tetramer unlikely due to steric hindrance (Fig 3).

Significantly reduced specific activity (Fig 5), decreased thermal stability (Fig 4) and a shift to a lower oligomeric state (Table 3) observed for *Pf*MDH-V190W and *Pf*MDH-E18W mutants suggest that tetrameric assembly of *Pf*MDH is crucial for its catalytic activity, although the AC and AB interfaces are well separated from the active sites. Furthermore, no obvious electrostatic connection can be observed between AC interface and the closest active site. Our kinetic data suggest that this is not as a result of the V190W or E18W mutations creating misfolded species, as the both mutants were still able to bind cofactor NADH and oxaloacetate, although with significantly altered affinities (Table 3). Interestingly, V190W mutation disrupting AC interface resulted in approx. 3x reduced affinity towards NADH and no apparent change in oxaloacetate binding (Table 3), while E18W showed approx. 10x reduced affinity towards NADH and 6x increased affinity towards oxaloacetate. These findings suggest different control mechanisms, facilitated by the proper formation of AB or AC interface.

As anticipated, stabilization of AB interface with additional hydrogen bonds (E18Q) improved NADH and oxaloacetate binding approx. 50-fold and 5-fold, respectively (Table 3). None of the *Pf*MDH variants (including wild type) addressed in this study showed measurable binding of NAD^+^ or malate, supporting the assumption that malate-forming reaction is more favored under standard conditions [24]. Furthermore, no significant thermal stabilization by NAD^+^ or malate could be observed by TSA (Table 3). These finding reflect the changes in catalytic activity, observed for all three mutants (Fig 5). Indeed, at intracellular malate concentration (approx. 1.6 mM), previously estimated by NMR spectroscopy [35], approx. 30x and 190x reduced specific activity for malate oxidation was observed for V190W and E18W mutants, respectively (Fig 5c). However, while V190W mutation 4x decreased specific activity for oxaloacetate reduction at sub-millimolar oxaloacetate concentration (0.625 mM), E18W mutant was approx. twice more active, compared to the wild type (Fig 5d). Similarly, in the same conditions E18Q stabilized mutant did not show significant changes in forward activity (malate–oxaloacetate, Fig 5c), while reverse reaction (oxaloacetate—malate) was 10x more rapid (Fig 5d).

These data also correlate with previous studies on *C*. *aurantiacus* MDH, where mutagenic alteration of the electrostatic interactions contributing to both AB and AC interfaces lead to pH-dependent thermal destabilization and up to 4-fold activity loss of resulting mutants compared to the wild type [36]. Furthermore, mutagenic disruption of AB interface in *E*. *coli* MDH using steric clashes resulted in monomeric species and dramatically decreased activity [37].

Our data then show that incorporation of *Pf*MDH-V190W into the wild-type assembly is possible *in vitro*, as co-purification using sequential affinity chromatography steps results in a sample that contains both wild-type and V190W *Pf*MDH species, whereas the control experiments did not (Fig 6). No measurable activity could be observed for this chimeric assembly *in vitro* (Fig 5c and 5d), demonstrating that incorporation of *Pf*MDH-V190W into the wild-type assemblies can provide a mechanism to specifically target the activity of *Pf*MDH. This is highly likely to be due to a perturbation of the oligomeric state through the introduction of a steric clash on the AC interface. Due to the activation effect observed for E18W mutant at sub-millimolar oxaloacetate concentrations we did not attempt to incorporate *Pf*MDH-E18W into the native assembly, as the aim of this study was rather specific inhibition.

The ability of *Pf*MDH-V190W to insert itself into the wild-type assembly and perturb its activity is of key importance for our future experiments, in which we will introduce this mutant as a recombinantly expressed protein within blood stage parasite cultures in order to provide validation of *Pf*MDH as a novel potential antimalarial drug target [4, 10]. By using controlled overexpression of the inactivated mutant within the parasite (using a transfection technique [38–41]), we propose to achieve overpopulation of the endogenous target protein and ensure that at least one mutant copy is present in each target protein assembly, thus rendering the target protein inactive. Our approach would potentially bypass the limitations often associated with conventional drug target validation techniques, such as poor inhibitor transportation, degradation, insufficient specificity or uncertainty in essential gene analysis [4]. The work presented here also demonstrates that oligomeric state control may have significant potential in validating other drug targets in the future, both through inhibition and stimulation of activity.

## Materials and methods

### Protein production and purification

Cloning, recombinant expression and purification of *Pf*MDH were performed according to previously reported manuscript[23]. Site-directed mutagenesis was performed using sequence specific primers and pASK-Iba3-*Pf*MDH-WT plasmid as a template (Table 4). Resulting plasmids pASK-Iba3-*Pf*MDH-V190W, pASK-Iba3-*Pf*MDH-E18W and pASK-Iba3-*Pf*MDH-E18Q encoded full length *Pf*MDH with C-teminal His_6_-tag and V190W, E18W and E18Q mutations, respectively. All constructs were verified by automated sequencing (Sanger). The mutant versions of *Pf*MDH were expressed and purified according to the same protocol as the wild type, with minor modifications.

**Table 4:** Primers used in this study.

Primer	Sequence
*Pf*MDH cloning for recombinant expression (pASK-IBA3, His_6_-tag)	
IBA3-MDH-s (BsaI, His_6_)	5’-GCGCGC**GGTCTC**CAATGACTAAAATTGCCTTAATAGGTAGTGGTC-3’
IBA3-MDH-as (BsaI, His_6_)	5’-GCGCGC**GGTCTC**AGCGCTTTAATGATGATGATGATGATGGCCTTTAATTAAGTCGAAAGCTTTTTGTGTG-3’
*Pf*MDH cloning for recombinant expression (pASK-IBA3, Strep-tag)	
IBA3-MDH-s (SacII)	5’-ATATCCGCGGATGACTAAAATTGCCTTA-3’
IBA3-MDH-as (NcoI)	5’-AGAGCCATGGCTTTTAATTAAGTCGAAAGC-3’
*Pf*MDH V190W Site-directed mutagenesis primers (pASK-IBA3)	
MDH-V190W-s	5’-GATATACATCGGTAAATGGTTGGCCTTTATCTGAATTTGTC-3’
MDH-V190W-as	5’-GACAAATTCAGATAAAGGCCAACCATTTACCGATGTATATC-3’
*Pf*MDH E18W Site-directed mutagenesis primers (pASK-IBA3)	
MDH-E18W-s	5’-CAAATCGGAGCAATTGTTGGATGGTTGTGTTTGCTGGAAAATCTTGG-3’
MDH-E18W-as	5’-CCAAGATTTTCCAGCAAACACAACCATCCAACAATTGCTCCGATTTG-3’
*Pf*MDH E18Q Site-directed mutagenesis primers (pASK-IBA3)	
MDH-E18Q-s	5’-CAAATCGGAGCAATTGTTGGACAATTGTGTTTGCTGGAAAATCTTGG-3’
MDH-E18Q-as	5’-CCAAGATTTTCCAGCAAACACAATTGTCCAACAATTGCTCCGATTTG-3’

Recognition sites for restriction enzymes used (specified in the primer name) are highlighted in bold. Mutations sites are underlined.

Briefly, after Ni-NTA purification, *Pf*MDH-V190W, *Pf*MDH-E18W, *Pf*MDH-E18Q and wild type samples were applied onto S200 10/300 size exclusion column (GE Healthcare), previously equilibrated with 100 mM Na-Phosphate pH 7.4 and 400 mM NaCl using NGC chromatograph (BioRad). The proteins eluted as single peaks with retention volumes of 18.4 mL (V190W), 17.8 mL (E18W), 16.8 mL (E18Q) and 17 mL (WT).

### Thermal Stability Assay (TSA)

TSA assays were performed in 96-well PCR plates (VWR) using SFX96 Real-Time PCR reactor (BioRad). The protein stability was assayed based on the increased fluorescence of the dye upon binding to the hydrophobic core of the unfolded protein. SYPRO Orange dye (5000 stock, Invitrogen) was added to the protein sample at 2 mg ml^-1^ in a 1:500 ratio. The protein samples were assayed against various buffer systems (pH gradient) as well as buffer components [30, 31]. The volume of each sample was 50 μL, the final protein concentration in each assay was 0.2 mg ml^-1^ (5,7 μM). Melting curve (in terms of increased fluorescence) of each sample was plotted against the temperature gradient (293–373 K) and the temperatures of the inflection points (T_m_’s) were used as indicators of the thermal stability of each sample.

### Determination of oligomeric state

The oligomeric state of *Pf*MDH wild type and its mutants was determined by static light scattering experiments performed inline with size exclusion chromatography using NGC (BioRad). *Pf*MDH-WT sample, purified to homogeneity and concentrated to 1.0 mg ml^-1^, was injected onto Superdex S75 10/300 (GE Healthcare) size exclusion column in line with MiniDAWN TREOS (Wyatt) three angle static light scattering device. The size exclusion column was previously equilibrated with 100 mM Na-Phospate pH 7.4, and 400 mM NaCl. An inlet filter was used to prevent big aggregates (>100 nm) from interfering with the measurements. Static light data was analysed using software provided by the manufacturers (ASTRA 6.1.5.22; Wyatt Technologies). The *Pf*MDH-WT sample eluted as a single peak and was characterized as a monodisperse (Mw/Mn = 1.002) tetramer with Mw of 140.5 ± 4.2 kDa (Table 3). The protein concentration and particle size were calculated based the protein theoretical absorbance at 280 nm [Abs 0.1% (1 mg ml^-1^) = 0.39; http://web.expasy.org/protparam]. Similarly, *Pf*MDH-E18Q sample was characterized as monodisperse (Mw/Mn = 1.003) tetramer with Mw of 139.4 ± 0.2 kDa (Table 3). The calculation of the extinction coefficients of *Pf*MDH-WT and E18Q samples was performed with 10% uncertainty, as the neither WT nor E18Q sequence contain tryptophan residues.

V190W and E18W samples were characterized as monodisperse dimers (Mw/Mn = 1.003) with calculated Mw of 70.5 ± 0.3 kDa and 76.6 ± 0.4 kDa, respectively (Table 3).

### Activity assays

The kinetic parameters of *Pf*MDH-WT, as well as *Pf*MDH-V190W, *Pf*MDH-E18W and *Pf*MDH-E18Q mutants were assayed in 100 mM Na-Phosphate pH 7,4, 400 mM NaCl. Specific activity of *Pf*MDH mutants was assayed based on the increased absorbance of NADH oxidized (NAD^+^ reduced) at 340 nm and measured as μmol NADH converted per minute by 1 mg of the enzyme. The reactions were performed in 1 ml cuvettes (Sarstedt) at room temperature using Jasco 650 UV-VIS spectrophotometer (Jasco GmbH). The forward reactions were performed using 50 nM enzyme pre-incubated in the assay buffer supplemented with 5 mM NAD^+^. The reactions were initiated using decreasing concentrations of DL-malate starting at 10 mM. Similarly, the reverse reaction of the reduction of oxaloacetate was performed by 50 nM of the enzyme in presence of 0.5 mM NADH and initiated by addition of oxaloacetate (highest concentration 10 mM). All measurements were performed in triplicates. No spontaneous NADH oxidation or NAD^+^ reduction in presence of the high substrate concentrations and absence of the enzyme was observed.

### Microscale Thermophoresis (MST)

MST measurements were performed on a Nanotemper Monolith NT.115 instrument (Nanotemper Technologies, GmbH) using His-tag fluorescent labeling. Each *Pf*MDH (WT or mutant) sample, purified to homogeneity, was freshly labeled with the Monolith His-Tag RED-tris-NTA labeling dye according to the supplied protocol (Nanotemper Technologies, GmbH). Measurements were performed in 100 mM Na-Phosphate pH 7,4 and 400 mM NaCl containing 0.05% Tween-20 in standard treated capillaries (MO-K002, Nanotemper Technologies, GmbH). The final concentrations of either labeled protein in the assay were 50 nM. The ligands (DL-malate, Oxalacetate, NAD+ and NADH) were titrated in 1:1 dilutions following manufacturer’s recommendations. All binding reactions were incubated for 10 min at room temperature followed by centrifugation at 20,000 x g before loading into capillaries. All measurements were performed in triplicates using automatically assigned LED power and medium MST power, LaserOn time was 30 sec, Laser Off time 5 sec.

### Co-purification of *Pf*MDH-WT and *Pf*MDH-V190W

Wild type *Pf*MDH open reading frame was re-cloned into pASK-IBA3 using a modification of the protocol described in [12, 23] (Table 4). Resulting plasmid encoded full-length *Pf*MDH-WT with C-terminal Strep-tag. Expression of both Strep-tagged *Pf*MDH-WT and His_6_-tagged *Pf*MDH-V190W mutant was performed as described above. The lysates were separately clarified by centrifugation, mixed and incubated for 2 hours at 277 K [42]. The subsequent co-purification from the mixed lysates was performed via the Strep-tactin as well as via Ni-NTA agarose (IBA Lifesciences, Qiagen).

Co-purified *Pf*MDH-WT and *Pf*MDH-V190W were visualized by western blot using a monoclonal Strep-tag II antibody (IBA) or anti-His antibody (Pierce, USA) and a secondary anti-mouse horseradish peroxidase labeled goat antibody (BioRad, Germany) as described in [42] (Fig 6).

### Crystallisation, X-ray data collection and structure determination

*Pf*MDH was purified to homogeneity, concentrated and crystallised as described in [12, 23]. The data collection statistics are shown in Table 1.

Crystal structure of *Pf*MDH was initially solved by molecular replacement using BALBES software [43] within the CCP4 suite [44] using the data collected on X13 beamline (EMBL, Hamburg). The PDB model of *Cryptosporidium parvum* MDH (**2HJR**) [27] was used as a search model [28], yielding a clear solution for 16 molecules in the asymmetric unit. The data from BM14 beamline (ESRF, Grenoble) was subsequently used for molecular replacement using MOLREP [45] and confirmed the previously identified solution supporting the presence of four tetrameric *Pf*MDH assemblies in the asymmetric unit. Rebuilding and refinement were carried out using Coot [46] and Refmac5 [47], respectively. The presence of non-crystallographic symmetry was used as a restraint in refinement at all stages. The final model refined against data to a resolution of 2.4 Å and has been deposited with the PDB [25] under the accession number **5NFR**. For more detailed data refinement statistics of *Pf*MDH please refer to Table 1

## Supporting information

S1 Data The study’s relevant raw MST data. (ZIP)
